# Influence of Surface Roughness on the Dynamics and Crystallization of Vapor-Deposited Thin Films

**DOI:** 10.1021/acs.jpcb.2c04541

**Published:** 2022-09-28

**Authors:** Aparna Beena Unni, Roksana Winkler, Daniel Marques Duarte, Katarzyna Chat, Karolina Adrjanowicz

**Affiliations:** † Institute of Physics, University of Silesia, 75 Pulku Piechoty 1a, 41-500 Chorzow, Poland; ‡ Silesian Center for Education and Interdisciplinary Research (SMCEBI), 75 Pulku Piechoty 1a, 41-500 Chorzow, Poland

## Abstract

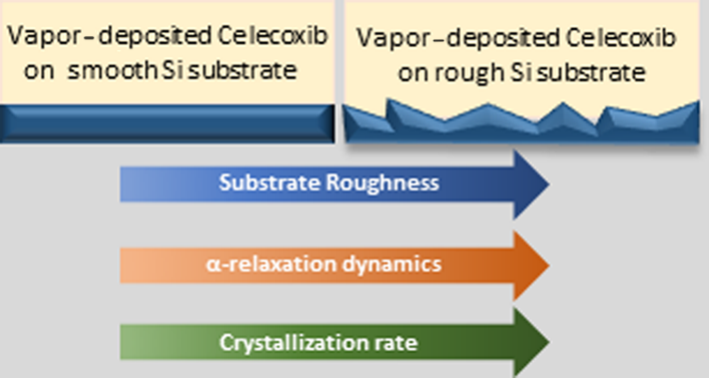

The substrate roughness is a very important parameter that can influence the properties of supported thin films. In this work, we investigate the effect of surface roughness on the properties of a vapor-deposited glass (celecoxib, CXB) both in its bulk and in confined states. Using dielectric spectroscopy, we provide experimental evidence depicting a profound influence of surface roughness on the α-relaxation dynamics and the isothermal crystallization of this vapor-deposited glass. Besides, we have verified the influence of film confinement on varying values of surface roughnesses as well. At a fixed surface roughness value, the confinement could alter both the dynamics and crystallization of vapor-deposited CXB.

## Introduction

The ultra-stable glasses (USGs), the nanometric confinement, and the surface roughness, all these three topics got discrete attention and are commendably discussed in the scientific community due to their significance in the design of novel devices. This article deals with the conjunction of these three critical topics.

At the outset, the vapor deposition technique got wide attention due to its ability to produce glasses with remarkable energetic and kinetic stability as well as very high densities.^1−3^ When deposited under appropriate conditions, they attain a near-equilibrium packing due to reduced moving restrictions.^4^ Thus, they possess a stable state, which can take millennia for the ordinary glass counterparts to acquire.^5^ Hence, the glasses produced by the vapor deposition technique under optimal deposition conditions are generally termed as USGs/superaged glasses. Such USGs are found to have a lot of technological applications, such as in the fabrication of organic light-emitting diodes,^6−8^ organic field-effect transistors,^9,10^ organic photovoltaic cells,^11−14^ and so forth.

Considering the nanometric confinement, this is a sought-after condition while dealing with the fabrication of miniaturized devices. However, when the materials are confined by their thickness, they show a lot of anomalous behavior compared to their bulk counterparts. This can include various properties such as its density,^15−17^ dynamics,^18−20^ crystallization,^21−23^ stability,^24,25^ and so forth. This is where the challenges in the practical implementation of a confined system lie. Many studies try to understand the underlying phenomena and optimize the material properties in a confined state. However, even after years of research, many of these topics are still intractable.

Finally, focusing on the surface roughness, the properties of materials under confinement are reported to be altered by the roughness of the substrate on which they are deposited. The surface roughness can directly influence the properties such as the adhesion,^26−28^ hydrophobicity,^29,30^ elastic modulus,^31^ electronic, magnetic, optical properties,^32−36^ and a lot more. Thus, considering its practical applications, it is inevitable to understand the surface roughness’s influence on the properties of thin films.

A study investigating how the surface roughness of the substrate can influence the properties of vapor-deposited glasses does not exist in the literature so far. In this work, we investigate the effect of surface roughness on the properties of vapor-deposited glasses in bulk as well as in 1D nanometric confinement. We investigate the α-relaxation dynamics and the crystallization behavior of films deposited on substrates with varying values of surface roughness, at a constant value of film thickness. In this way, the influence of substrate roughness on the confined vapor-deposited glasses could be understood better. On the other hand, we also study the α-relaxation dynamics as well as the crystallization behavior at fixed values of surface roughnesses by varying the thickness of the USGs. This study can help us understand how the confinement influences the properties of USGs at a constant value of surface roughness ranging from 0.5 to 5 nm. Thus, this article investigates the influence of surface roughness and the confinement effects on the properties of vapor-deposited glasses.

## Materials and Methods

**Figure 1:**
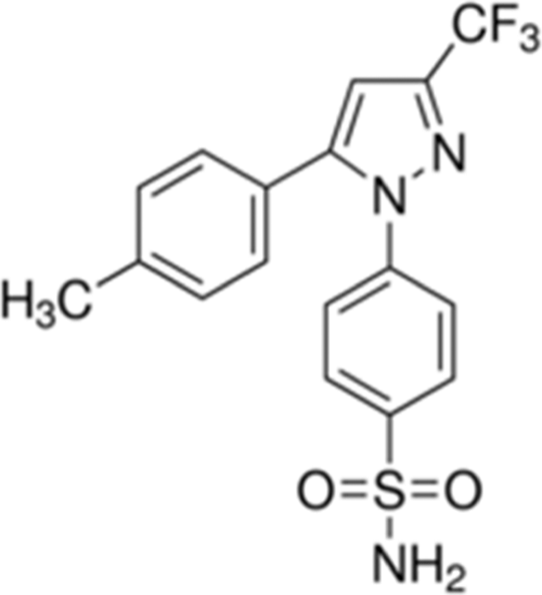
The molecular structure of CXB compound investigated in this study.

In this work, we study a molecular drug celecoxib (CXB) vapor-deposited on top Si wafers with varying surface roughness values. CXB, with a molecular weight of 381 g/mol, was supplied from Polpharma (Starogard Gdanski, Poland). The material was provided as a white crystalline powder. The molecular structure of CXB is shown in Figure 1.

The melting point of the as-received crystalline material is estimated as *T*_m_ = 435 K by means of differential scanning calorimetry. The glass-transition temperature, *T*_g_ = 326.6 K, was obtained by quench-cooling the bulk sample with a 10 K/min heating scan. The melting and the glass-transition values are in well agreement with the literature data.^37^ The details of the DSC thermogram for bulk CXB are given in our previous article.^38^ Conductive silicon wafers (purchased from SIL’TRONIX, France) with a native oxide layer are used as the substrate. The wafers are oriented in (1 0 0) and possess a resistivity value from 0.001 to 0.003 Ω cm. This also acted as the lower electrode for the dielectric measurements. Before vapor deposition, the wafers were cleaned with air plasma (using Henniker Plasma HPT-100) to remove the organic contaminants from the surface. To vary the surface roughness of such Si wafers, they were exposed to the vapors of hydrofluoric acid (HF) for different amounts of time. The HF treatment helps to preserve the chemical identity of the substrate surfaces.

**Figure 2:**
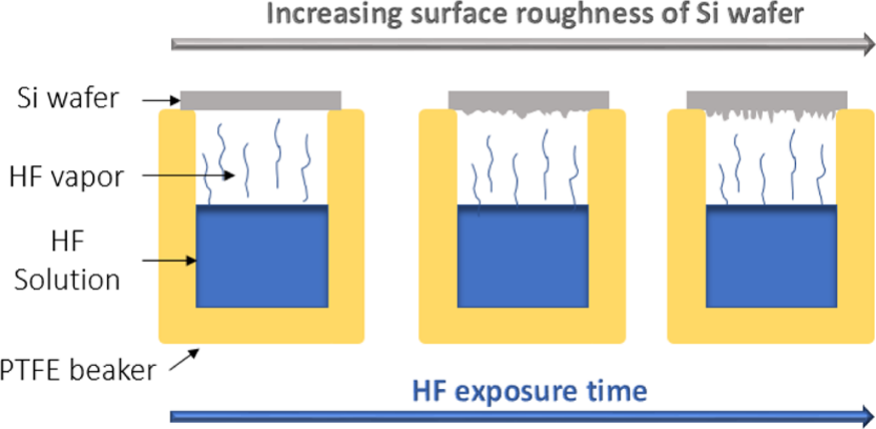
Schematic diagram showing the experimental setup used to modify the surface roughness of the Si wafers. The wafers were exposed to HF for different amounts of time to obtain various surface roughness values.

The HF was purchased from Sigma-Aldrich, which possesses a 48 wt. % in water. The Si wafers were placed atop the PTFE (Teflon) beakers containing a constant volume of HF solution, as shown in the schematic diagram Figure 2. The etching mechanism occurring in such a system is well explained in the literature.^39−41^ Similarly, we modified the surface roughness by controlling the time of exposure of the Si wafers to HF vapor. Further measurements and analysis were done at different locations of the same sample as well as on multiple samples to ensure reproducibility and precision. The surfaces after HF exposure were thoroughly cleaned with deionized water and were dried under ambient atmospheric conditions for 2 days before the deposition process. This ensures that the hydrogen-terminated surface is oxidized back to a hydroxyl-terminated Si wafer.^39,42^ Thus, the deposition is done on −OH-terminated Si surfaces with varying surface roughness values.

The physical vapor deposition (PVD) technique was used for depositing CXB on Si wafers. The films were deposited in an ultra-high-vacuum chamber with a base pressure between 10^–7^ and 10^–8^ Torr. The substrate was kept on a temperature-controlled stage and was kept at 0.85*T*_g_ of CXB during the deposition process. The CXB, loaded in an alumina thermal crucible, was heated inside the vacuum chamber for deposition. The deposition rate was around 0.2 nm/s measured in situ during evaporation by the quartz crystal microbalance (QCM), which matches with the rate calculated by the estimated thickness and the time taken for the deposition process. The thickness of the deposited layer measured by QCM is also compared with the atomic force microscopy (AFM) results, which was measured by making a scratch using a soft pen on the film and measuring the height of the step using JPK’s NanoWizard 3 NanoScience atomic force microscope. The measurements were done in tapping mode using a silicon cantilever and were analyzed using Gwyddion and WsXM software. The thickness of the obtained films was also reconfirmed using a spectroscopic ellipsometer (Semilab SE-2000 spectrometer). The measurements were done at incident angles of 65, 70, and 75° at ambient conditions. A multilayer model consisting of the Si substrate, native oxide layer, and CXB was considered for the analysis.

The dielectric measurements were performed using a high-resolution Alpha Analyzer assisted by a Quatro temperature controller (both from Novocontrol Technologies GmbH) in the frequency range from 10^–1^ to 10^6^ Hz at temperatures varied at 5 or 10 K steps. The data were acquired using a nanostructured Si electrode (1 × 1 mm nanostructured die with highly insulating square SiO_2_ spacers of 5 μm side length and 60 nm height) as the upper/counter electrode and the conducting Si substrate as the lower electrode and the film measured acted as the dielectric. The configuration and details of the model electric circuit for the considered system geometry are discussed in detail in our previous work^19^ and the experimental details as well as the details of data analysis are given in the Supporting Information.

## Results and Discussion

### Characterization of Rough Surfaces

**Figure 3:**
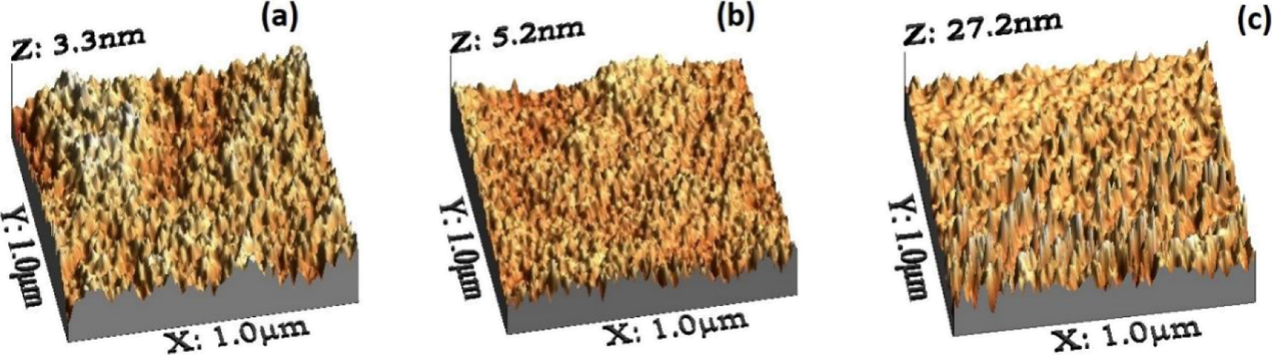
AFM 3D topographic images showing Si substrates treated with HF for different amounts of time, (a) 20, (b) 40, and (c) 60 min, which produced varying surface roughnesses *R*_rms_ ∼ 0.5 nm, *R*_rms_ ∼ 1.5 nm, and *R*_rms_ ∼ 5 nm, respectively.

In order to investigate the influence of surface roughness, we have modified the roughness of silicon surfaces by HF treatment. The surfaces with modified roughnesses are characterized using atomic force microscopy. Figure 3 shows the representative 3D AFM topographies of Si surfaces with varying roughness values for a scanning range of 1*1 μm.

We have obtained an increased value of roughness with increasing time of exposure of silicon wafers to the HF, which is in line with the observations of Huang et al.^39^ The rms roughness values of 0.5, 1.5, and 5 nm were obtained, respectively, for the HF exposure times of 20, 40, and 60 min. Although we could obtain statistical surface roughness parameters from direct AFM image analysis, these values are highly reliant on the scan rate, scales, resolution, measurement specifics, and so forth.^43,44^ Hence, we additionally perform fractal geometrical analysis using power spectral density (PSD) functions by a fast Fourier transform algorithm. In this method, the AFM images are represented by the spectral strength densities over a wide range of distinct spatial frequencies. Hence, it is possible to clearly understand the magnitude and the significance of surface imperfections with different spatial frequencies. Since the surface morphology of the films consists of two-dimensional coordinates with height values, we have used a two-dimensional discrete PSD function as follows^33,45^
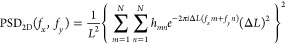
where *L*^2^ is the scanned surface area, *N* is the number of data points per line and row, *h*_*nm*_ is the profile height at position (*m*,*n*), *f*_*x*_ and *f*_*y*_ are the spatial frequency in the “*x*” and “*y*” directions, and Δ*L* is the sampling distance (Δ*L* = *L*/*N*).

**Figure 4:**
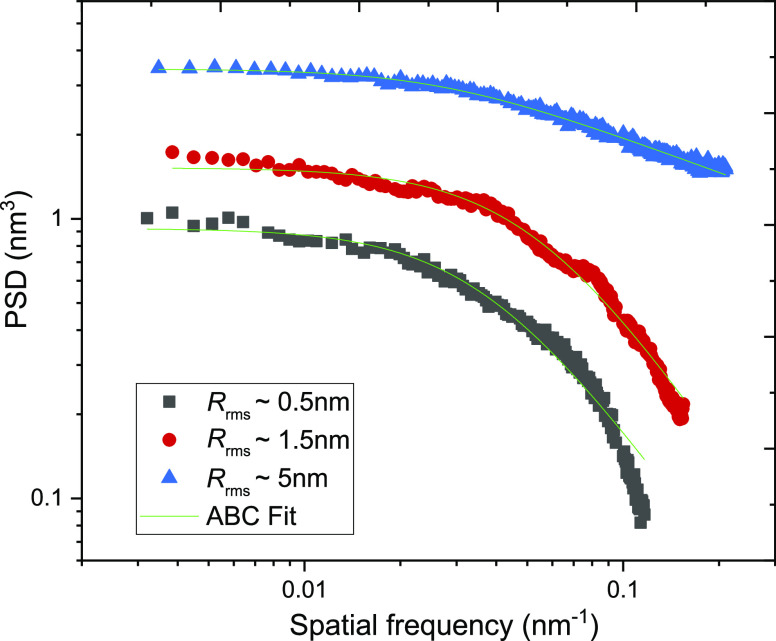
PSD profile of a Si wafer surface with *R*_rms_ ∼ 0.5, 1.5, and 5 nm, respectively (20, 40, and 60 min of HF treatment). The green line represents the ABC fit for the respective data.

Figure 4 shows the PSD profile of the Si wafer surface with varying roughness values and their ABC model fits. The ABC/*k* correlation model explains the random distribution of topographic characteristics and allows the quantitative comparison between samples over large length scales. The following equation represents the ABC model of PSD^46^
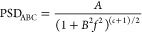
where *A* is the shoulder parameter, which is the value of the spectrum in the low-frequency limit, *B* is the correlation length, which sets the point of the transition between the low- and high-frequency behavior, and *C* is the exponent of the power-law falloff at high frequencies. From the results shown in Figure 4, one can see an increase in the value of *A* with an increasing roughness value. There is no commendable variation in the slope or the knee that separates the low and high frequency with changing roughness. The significant variation is observed only for the height in the *Z* direction, and these curves represent self-affine, randomly rough surfaces.

### α-Relaxation Dynamics of Vapor-Deposited CXB Films on Si Substrates with Varying Values of Surface Roughness

**Figure 5:**
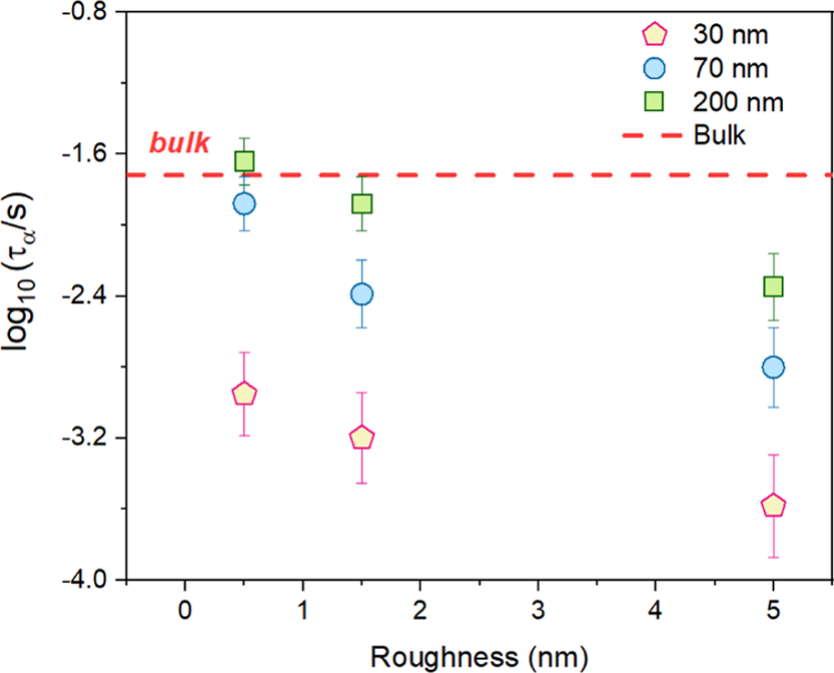
Mean α-relaxation time (τ_α_) plotted as a function of the roughness of the silicon substrate for vapor-deposited CXB films of thicknesses of 30, 70, and 200 nm.

Recent studies by Fiori et al. using X-ray scattering and spectroscopic ellipsometry indicated that the substrate exerts negligible influence on the structure of PVD glass.^47^ A modified molecular packing was observed at a length of ∼8 nm near the substrate by the molecular dynamics simulations, which agrees with the grazing incidence X-ray scattering results.^48^ On the other hand, the studies by Yokoyama et al. propose a commendable influence of the underlying substrate (even for ∼100 nm thick films) on the structure of PVD glass films.^7,49^ It also points that the substrate roughness can influence the structure of vapor-deposited glasses. The significance of such studies lies in the fact that the structure and orientation of vapor-deposited glasses can play a crucial role in determining their properties and thereby their practical functionality. Here, we modify the roughness of the substrate to investigate whether it can influence the dynamics of a PVD glass, CXB. The measurements were carried out on confined and bulk vapor-deposited CXB films deposited on Si wafers with varying roughness values. Figure 5 shows the mean α-relaxation time plotted as a function of the surface roughness of the substrate.

The dynamics of 30 nm films are observed to be faster on the rougher substrate compared to the smoother one. A similar trend is observed for 70 nm and the bulk films. Thus, irrespective of film thickness, there is a systematic increase in the dynamics with increasing values of the substrate roughness.

In order to understand this observation, we try to calculate the interfacial energy between CXB and SiO_2_. Their surface free energy values are given in Table 1. The total surface energy γ^total^ of a sample is expressed by γ^total^ = γ^D^ + γ^P^, where γ^D^ is the dispersive component and γ^P^ is a polar component of the surface energy, respectively.^52^

**Table 1:** Total Surface Energy γ^total^ and Its Dispersive γ^D^ and Polar γ^P^ Components for CXB on the SiO_2_ Surface^50,51^

material	γ^total^ (mJ m^–2^)	γ^D^ (mJ m^–2^)	γ^p^ (mJ m^–2^)
CXB	50.0	45.2	4.8
SiO_2_ surface	47	44.6	2.3

From this, one can estimate the interfacial energy between CXB and the SiO_2_ using the Fowke’s rule as follows^53^
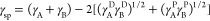
where, in our case, *A* and *B* refer to the substrate and CXB, respectively. The interfacial energy between CXB and SiO_2_ is thus calculated to be 0.55 mJ m^–2^. In general, if the γ_SP_ < 2 mJ m^–2^, a depression of the glass transition temperatures should be observed, while for γ_SP_ > 2 mJ m^–2^, an increase of *T*_g_ should be observed.^51^

Tsui et al. have experimentally shown that the glass-transition temperature of polymer films decreases compared to the bulk values at low values of the interfacial energy.^54^ In line with this, a faster chain dynamics was observed for a weakly adsorbing polymer on a substrate by Ayalur-Karunakaran et al.^55^ Analogously, in our case, due to the comparatively weak interaction of the molecule with the substrate, one can expect less packing density of molecules near the supporting substrate interface than for a stronger interaction case. Hence, as the roughness increases, the incomplete filling of the asperities can be due to the weak interactions of the material with the substrate. A very similar case is reported on aluminum substrates with varying roughness values, where the authors observed enhanced segmental dynamics for poly 4-chlorostyrene (P4ClS) thin films with increasing roughness values.^56^ On the other hand, our group has reported a decrease in the segmental dynamics of the same polymer with an increasing value of surface roughness.^19^ The key difference between these two observations is that, in the former case, P4ClS is deposited on an aluminum substrate which possesses comparatively weak interaction with the polymer (∼0.11 mJ m^–2^), and conversely, the P4ClS-silicon surface has a stronger interaction of around 4 mJ m^–2^. In the current study, the vapor deposition in rougher substrates could accelerate the α-relaxation dynamics of the films as the interfacial interaction between the substrate and the coated material is weak. Further studies are required to scrutinize the effect of interfacial interaction strength on the properties of vapor-deposited thin films, which is beyond the scope of this work.

### α-Relaxation Dynamics of Confined Vapor-Deposited CXB Films at a Constant Value of Surface Roughness

**Figure 6:**
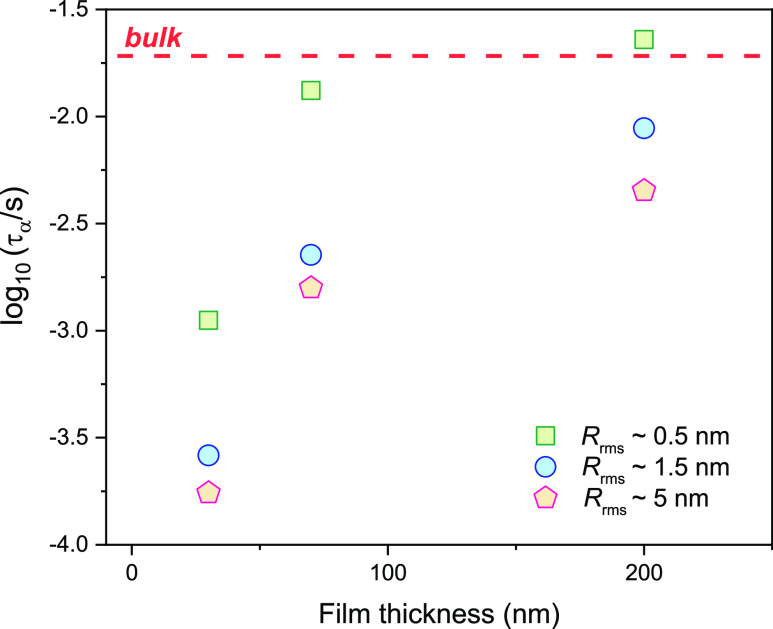
Mean α-relaxation time (τ_α_) plotted as a function of inverse temperature (*T*) for vapor-deposited CXB films with varying film thicknesses deposited on Si substrates with roughness values of (a) ∼0.5, (b) ∼1.5, and (c) ∼5 nm.

Figure 6 shows the thickness dependence of α-relaxation dynamics for vapor-deposited CXB films deposited on surfaces with different roughnesses. This is done to understand the confinement effects of a vapor-deposited film on rough surfaces.

Considering the films deposited on substrates with an rms roughness value of 0.5 nm, the 200 nm films show bulk-like dynamics and 70 nm films show a slightly faster dynamics. In contrast, the 30 nm film exhibits the fastest dynamics compared to the rest. Thus, the films deposited on substrates with *R*_rms_ = 0.5 nm show an increase in the dynamics with a decreasing film thickness value. A similar trend is observed for films confined at higher values of surface roughness as well. At lower surface roughness values, the 200 nm films show bulk-like dynamics, whereas at *R*_rms_ = 5 nm, slightly faster dynamics are observed compared to the bulk. Hence, as the thickness decreases, there is an increase in the α-relaxation dynamics of vapor-deposited films on rough substrates. The first experimental evidence for the size effects in the glass transition of thin films of an organic molecule grown from the vapor phase was given by Leon-Gutierrez et al.^57^ Using nanocalorimetry, they could observe a decrease in the onset of glass transition with decreasing film thickness. They also suggest that a faster dynamic influenced by the outer film surface aids the transformation of ultrathin vapor-deposited glasses into liquid. Computer simulation studies also hold up the observation of enhanced dynamics near the free surface of vapor-deposited glasses.^58^ Considering films under geometric confinement, the influence of the surface region will be much pronounced compared to bulk films. Our group has also reported a similar behavior for confined vapor-deposited films of CXB.^38^ We have observed faster α-relaxation dynamics for CXB films with decreasing thickness. However, in that case, CXB was deposited on the native silicon surface. Thus, the weak interfacial interactions together with the dynamics enhancement contributed by the free surface during confinement can explain the faster dynamics observed in confined vapor-deposited films. Hence, from this study, despite the roughness value being high or low, the α-relaxation dynamics of vapor-deposited films become faster when the films are confined by their thickness.

### Crystallization of Confined Vapor-Deposited Films of CXB on Si Substrates with Varying Values of Surface Roughness

The vapor deposition technique is reported to be a novel method that can slow down the crystallization of organic glasses.^59,60^ The reduction in the crystallization rate upon vapor deposition is observed both above and below the glass-transition temperature.^38,59,60^ Here, we investigate the influence of both confinement and roughness on the crystallization behavior of vapor-deposited CXB.

**Figure 7:**
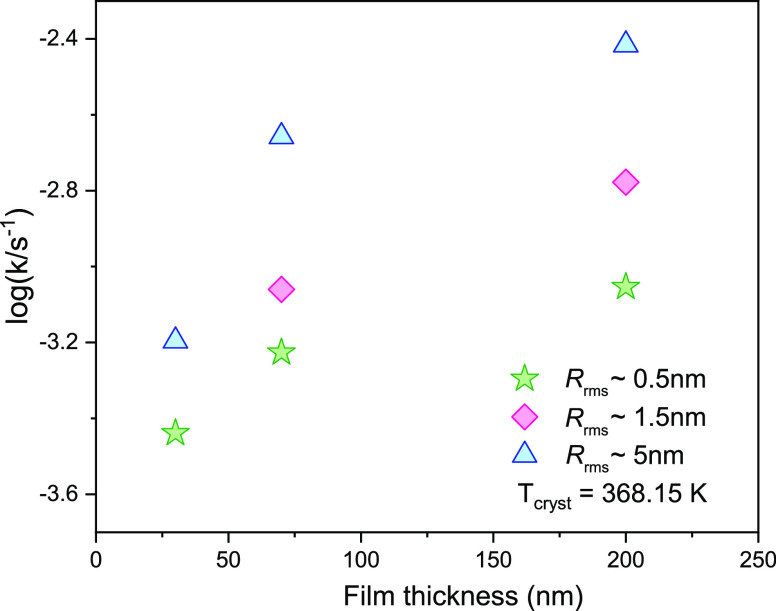
Crystallization rate *k* as a function of film thickness obtained for vapor-deposited CXB films on substrates with varying surface roughness measured at 368 K. The dashed lines are just a guide for the eyes.

In this work, we study the crystallization at 368.17 K, which was carefully chosen for the following reasons. The crystallization kinetics of CXB can take too long to be measured by dielectric spectroscopy at room temperature or at a temperature below its *T*_g_ value. Moreover, below *T*_g_, the alpha relaxation reflecting cooperative molecular movements is out of the experimental window. In such a case, only secondary relaxation processes can be seen. Besides, it is also important to ensure that the samples are not dewetted in these experiments. Doing experiments in higher temperatures can promote the dewetting in rough surfaces. Hence, a safe temperature value of 368.17 K was chosen so that the films are stable without dewetting, and at the same time, we can follow the alpha relaxation peak shifts. From Figure 7, one can observe the variations in the crystallization rate for films at a constant thickness (but varying values of surface roughness) as well as the films having the same surface roughness values (but varying values of film thickness). As shown in Figure 7, the rate of crystallization slows down at constant values surface roughness with decreasing film thickness. The trend is valid for all surface roughness values considered by us. On the other hand, at a constant value of film thickness (but at varying surface roughness values), the crystallization is accelerated with increasing surface roughness values. Focusing on the influence of film thickness on the crystallization rate, the theoretical analysis by Escleine et al. (later verified by computer simulations)^61^ on the influence of specimen thickness on isothermal crystallization kinetics has predicted that a decrease in the film thickness can result in slower crystallization kinetics and a decrease in the Avrami exponent.^62^ This is also in agreement with our experimental results. Moreover, many experimental investigations on confined films confirm that the crystallization slows down with the decreasing film thickness.^63−67^ Considering the influence of surface roughness on the crystallization, at a constant value of film thickness, we observe an increase in the crystallization rate with an increased surface roughness value. The studies by Yokoyama et al. showed that if the substrate surface is smooth enough, the coated material can easily migrate locally on the heated substrate and aggregate without crystallization.^7^ A comparative study on rough versus smooth surfaces was conducted based on the hydrothermal synthesis of WO_3_ films on rough as well as smooth surfaces.^68^ This study describes that nucleation mainly occurs at the site with an irregularity on the surface. The surface roughness could increase the heterogeneous nucleation and growth of crystals, leading to the increase in the degree of grain boundary at a high angle. An increased rate of nucleation with the increased surface roughness value was observed while conducting the crystallization experiments on metal surfaces that were ground to give different degrees of roughness values.^69^ The recent kinetic Monte Carlo simulation results also agree with these experimental observations.^70^ In agreement with this literature, one can assume that the surface roughness can enhance the heterogeneous nucleation, leading to a faster crystal formation in vapor-deposited films. Thus, the substrate roughness can accelerate the crystallization rate in vapor-deposited glasses.

## Conclusions

In this work, we have studied the dynamics and crystallization behavior of confined vapor-deposited films of CXB on Si wafers with varying surface roughness values. The fractal geometrical analysis using PSD functions revealed that our substrate surfaces are self-affined and randomly rough. We found that the α-relaxation dynamics of CXB films are faster on the substrates with a higher value of surface roughness. This result is rationalized by the weak interfacial interaction between the CXB and silicon substrate. Besides, when the thickness reduces at a constant surface roughness value, the dynamics speed up. This trend is observed for both higher and lower values of surface roughnesses. The isothermal crystallization studies showed that crystallization rates increase with the increasing values of surface roughness. At a constant roughness value, the crystallization rate is found to decrease with decreasing film thickness. Thus, this work scrutinizes the roughness effect on vapor-deposited films under 1D confinement. As the surface morphology and dimension can directly influence the functionality of films, this study can highly contribute to the design and engineering of vapor-deposited thin films with tailored properties.

## Supporting Information Available

Representative dielectric loss spectra of a CXB film on a Si wafer and crystallization measurements performed using dielectric spectroscopy (PDF)
