# Supplementary material for: Influence of Surface Roughness on the Dynamics and Crystallization of Vapor-Deposited Thin Films
Source: J Phys Chem B. 2022 Sep 28;126(40):8072–9. doi: 10.1021/acs.jpcb.2c04541 (PMC9574919; doi:10.1021/acs.jpcb.2c04541)
Supplement: Supplementary file 1 — jp2c04541_si_001.pdf [file jp2c04541_si_001.pdf]

Supplementary Material for

The influence of surface roughness on the dynamics and crystallization of a confined vapor deposited glass

Aparna Beena Unni,[†,‡] Roksana Winkler,[†,‡] Daniel Marques Duarte,[†,‡] Katarzyna Chat[†,‡] and

Karolina Adrjanowicz[†,‡]

[†] Institute of Physics, University of Silesia, 75 Pulku Piechoty 1a, 41-500 Chorzow, Poland

[‡] Silesian Center for Education and Interdisciplinary Research (SMCEBI), 75 Pulku Piechoty 1a, 41-500 Chorzow, Poland

S1: The representative dielectric loss spectra of celecoxib film on Si wafer

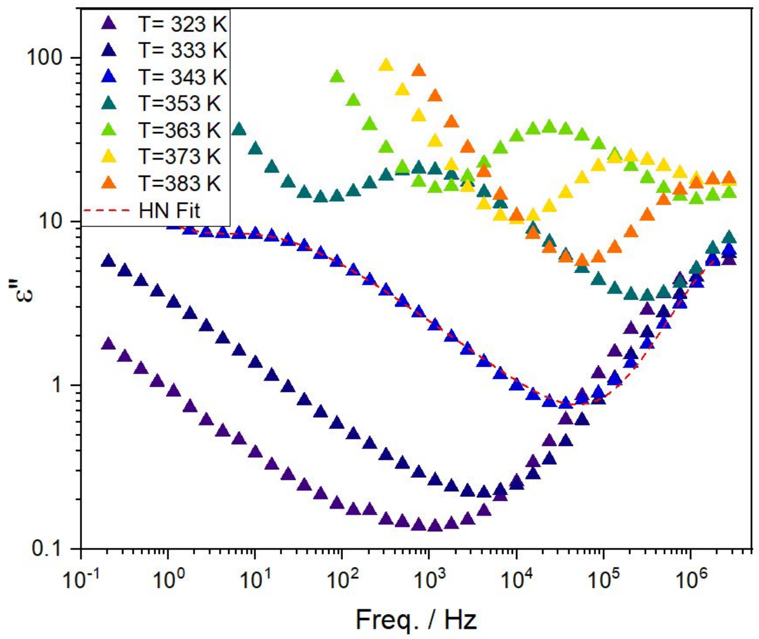

Figure S1. *Dielectric loss spectra of 70 nm CXB film vapor deposited on Si wafer with 0.5 nm roughness measured upon heating in the temperature range 323 K to 383 K*

In the figure S1, we show the dielectric loss spectra of a 70 nm thick celecoxib film on a Si wafer with a roughness value of 0.5 nm. The value of total permittivity ε_{tot} is fitted to the observed permittivity assuming that ε_{sam} is given by the sum of Havriliak-Negami (HN) function and a dc-conductivity term.[1]

$$\varepsilon_{sam}^{*}(\omega) = \varepsilon_{\infty} + \frac{\Delta\varepsilon}{\left[1 + (i\omega\tau_{HN})^{\alpha}\right]^{\gamma}} + \frac{\sigma_{dc}}{i\omega\varepsilon_0}\ , \qquad (1)$$

where $\Delta\varepsilon$ is the relaxation strength, τ_{HN} denotes the relaxation time, α and γ are the shape parameters, and σ_{dc} quantifies the level of conductivity. These parameters as well as the electrode resistance R are considered adjustable. The characteristic time constant τ_{HN} in the empirical HN function is related to the relaxation time at the maximum of the loss peak τ_{max} by the following relation

$$\tau_{max} = \tau_{HN} \sin^{-\frac{1}{\alpha}}\left[\pi\alpha\gamma/(2+2\gamma)\right] \sin^{\frac{1}{\alpha}}\left[\pi\alpha/(2+2\gamma)\right] \qquad (2)$$

The τ_{max} values are thus derived from these fits. In this figure, a representative HN fit is done for the loss peak at T= 343K which provided a τ_{max} value of 1.079 e^{-2} s. The mean α-relaxation time (logarithmic of the τ_{max}) is plotted as a function of temperature/ film thickness (figure 5, 6 in the main text) to obtain their respective influences.

S2: Crystallization measurements performed using dielectric spectroscopy

Considering the crystallization measurements by using dielectric spectroscopy, Figure S2. shows the evolution of the real and imaginary parts of the complex dielectric permittivity as measured at 368 K for a sample vapor-deposited CXB thin film.

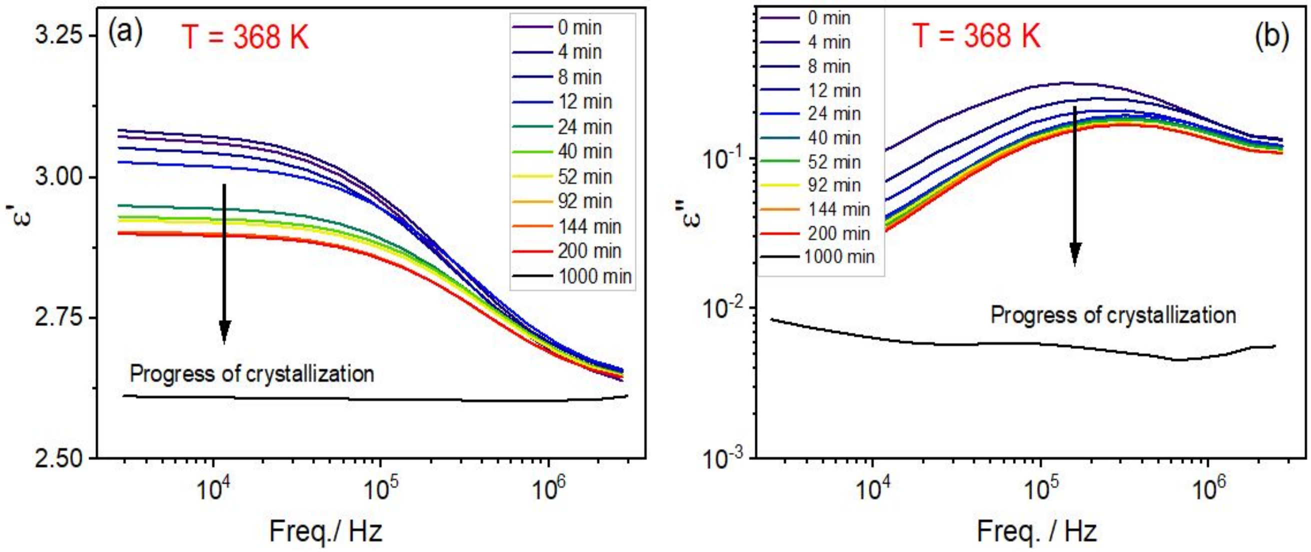

Figure S2. Changes in the (a) real and (b) imaginary parts of the complex dielectric permittivity (b) during the isothermal crystallization of vapor-deposited CXB thin film at 368 K.

An observed drop of the strength of the α-relaxation upon time-dependent measurements signifies crystallization progress. The crystallization kinetics was analysed by following changes in the dielectric permittivity for the selected frequency (static limit), which was normalized based on the following formula

$$\varepsilon'_N(t) = \frac{\varepsilon'(t=0) - \varepsilon'(t)}{\varepsilon'(t=0) - \varepsilon'(t=\infty)} \quad (3)$$

where ε′(t) is the value of the static dielectric permittivity for the selected frequency at a given time t, ε′(t = 0) is the value of the static dielectric permittivity for the same frequency chosen at the beginning of the crystallization, and ε′(t = ∞) is the long-time limiting value. The representative crystallization kinetic curves obtained for CXB vapor-deposited film is shown in Figure s3.

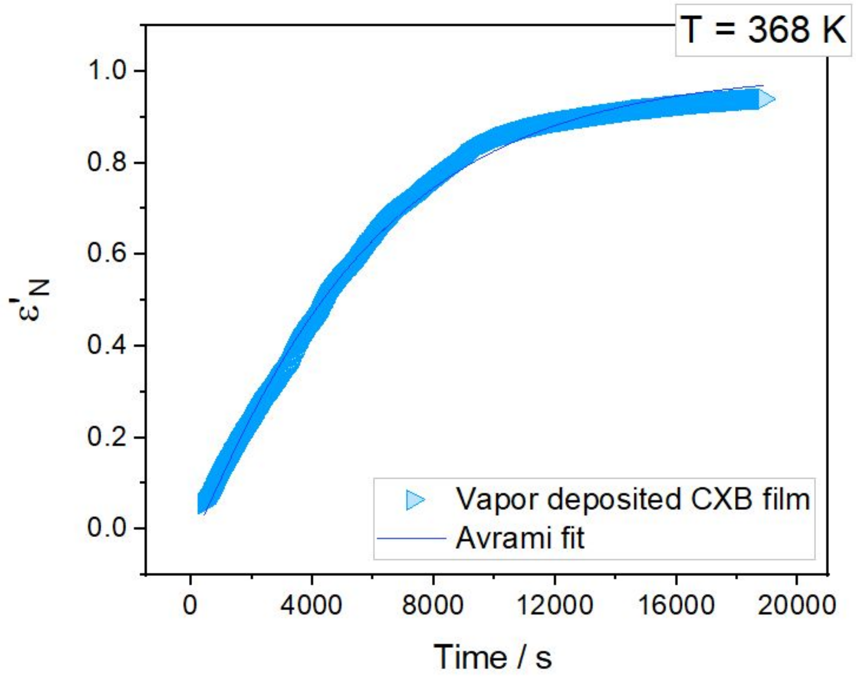

Figure S3. *Isothermal crystallization of vapor deposited CXB thin film at 368 K demonstrating the evolution of normalized dielectric permittivity with time. Solid line is the Avrami fit to the data.*

We find crystallization rate and Avrami parameter by fitting the data using the Avrami equation. Where, the transformed crystalline volume fraction can be described as

$$V \equiv \varepsilon'_N(t) = 1 - exp(-kt^n) \qquad (4)$$

where k is the rate constant, and n is the Avrami parameter. The crystallization rate provides combined information about the rate of nucleation (N) as well as the crystal growth (G), where $k = NG^{n-1}$.

References

(1) Beena Unni, A.; Chat, K.; Duarte, D. M.; Wojtyniak, M.; Geppert-Rybczyńska, M.; Kubacki, J.; Wrzalik, R.; Richert, R.; Adrjanowicz, K. Experimental Evidence on the Effect of Substrate Roughness on Segmental Dynamics of Confined Polymer Films. *Polymer.* **2020**, *199*, 122501. https://doi.org/10.1016/j.polymer.2020.122501.
